# Optimized Nb-Based Zeolites as Catalysts for the Synthesis of Succinic Acid and FDCA

**DOI:** 10.3390/molecules25214885

**Published:** 2020-10-22

**Authors:** Magdi El Fergani, Natalia Candu, Madalina Tudorache, Pascal Granger, Vasile I. Parvulescu, Simona M. Coman

**Affiliations:** 1 Department of Organic Chemistry, Biochemistry and Catalysis, Faculty of Chemistry, University of Bucharest, Bdul Regina Elisabeta 4-12, 030016 Bucharest, Romania; magdi.belkassim@drd.unibuc.ro (M.E.F.); natalia.candu@chimie.unibuc.ro (N.C.); madalina.sandulescu@g.unibuc.ro (M.T.); 2 University of Lille, CNRS, Centrale Lille, ENSCL, Univ. Artois, UMR 8181—UCCS—Unité de Catalyse et Chimie du Solide, F-59000 Lille, France; pascal.granger@univ-lille.fr

**Keywords:** zeolite, silicalite, niobium oxide, HMF, glucose, wet oxidation, organic peroxides, succinic acid, FDCA

## Abstract

Nb(0.05 moles%)-zeolites prepared via a post synthesis methodology (BEA, Y, ZSM-5), or a direct sol-gel method (Silicalite-1) were investigated in the hydroxymethylfurfural (HMF) oxidation by both molecular oxygen, in aqueous phase, and organic peroxides, in acetonitrile. The catalysts prepared through the post synthesis methodology (i.e., Nb-Y5, Nb-ZSM25, Nb-Y30, Nb-BEA12, and Nb-BEA18) displayed a mono-modal mesoporosity and contain residual framework Al-acid sites, extra framework isolated Nb(V)O-H and Nb_2_O_5_ pore-encapsulated clusters, while Nb-Sil-1, prepared through a direct synthesis procedure, displayed a bimodal micro-mesoporosity and contains only –Nb=O species. These modified zeolites behave as efficient catalysts in both HMF/glucose wet oxidation to succinic acid (SA) and HMF oxidation with organic peroxides to the 2,5-furandicarboxylic acid (FDCA). The catalytic behavior of these catalysts, in terms of conversion and especially the selectivity, mainly depended on the base/acid sites ratio. Thus, the HMF/glucose wet oxidation occurred with a total conversion and a selectivity to SA of 37.7% (from HMF) or 69.1% (from glucose) on the Nb-Y5 catalyst, i.e., the one with the lowest base/acid sites ratio. On the contrary, the catalysts with the highest base/acid sites ratio, i.e., Nb-ZSM25 and Nb-Sil-1, afforded a high catalytic efficiency in HMF oxidation with organic peroxides, in which FDCA was produced with selectivities of 61.3–63.8% for an HMF conversion of 96.7–99.0%.

## 1. Introduction

The recent interest for bio-plastics has raised the efforts for the production of dicarboxylic acids from biomass sources, thus completing their traditional uses in feed and food [1,2]. Among these, furan-2,5-dicarboxylic (FDCA) and succinic (SA) acids are extremely important monomers for these polymers [3,4,5,6,7]. However, the current approaches for the production of both FDCA and SA still suffer from a series of drawbacks such as harsh reaction conditions, metal salts as catalysts, and dangerous organic solvents that impose alternative sustainable pathways able to overcome these barriers. 

In this context, not long ago, we developed a highly efficient multi-functional Ru-based Fe_3_O_4_@SiO_2_ magnetic catalyst able to convert both levulinic acid (LA) (S_SA_ = 96–98% for X_LA_ = 59–79%) [8] and glucose to SA (selectivity of 87.5%, for a >99.9% conversion of glucose) [9] through a one-pot catalytic wet oxidation (CWO). The same catalyst affords an efficient oxidation of HMF to FDCA (selectivity of 80.6% to FDCA for a 92% conversion of HMF), as we recently demonstrated [10]. However, the CWO procedure requires the addition of *n*-butylamine in the liquid phase, which contravenes the green chemistry principles. Moreover, although the noble metal catalysts generally show high catalytic performances towards the oxidation reactions, their high cost hampers the large-scale production of these molecules. Such being the case, the development of heteroatomic Lewis acid- [11,12] and bifunctional-zeolite [13] catalysts through a zeolite framework dealumination followed by the insertion of a heteroatom in the obtained hydrophobic environment [14,15,16,17] may provide an innovative cheap alternative to the noble metals-based catalysts synthesis [18]. Such materials have already proved efficient for the production of different platform molecules [17,19,20]. 

Taking into consideration these statements, we focused our interest on a post synthesis strategy for the production of highly dispersed Nb species (0.02–0.05 moles%) into the dealuminated BEA-zeolite matrix [21]. The novel catalysts demonstrated a high catalytic efficiency for the glucose oxidation to SA. Thus, in the presence of the Nb(0.05)-BEA catalyst, SA was obtained with a selectivity of 84% for a total conversion of glucose. This catalytic performance was attributed to a concerted contribution of residual framework Al-acid sites and extra-framework isolated Nb(V)O-H and Nb_2_O_5_ pore-encapsulated clusters. On the other side, previous studies on the catalytic performance of niobia showed that the performance of these catalysts is closely related to the preparation route [22,23,24,25,26], the nature of precursors [27,28,29,30], and of the support [31,32,33,34]. Direct synthesis procedures for the niobium incorporation in mesoporous siliceous matrixes (i.e., MCM-41 or MCM-48) [35,36,37,38,39,40,41,42] led, for instance, to incorporated Nb^5+^ cations as isolated NbO_4_ species. Such species exhibit redox rather than acidic properties, therefore displaying unique oxidation properties. 

Taking into account the results already reported with Nb-based BEA zeolite catalyst [21] in the glucose oxidation and with the aim to extend the Nb-based zeolites catalysts applicability, we focused our efforts on the optimization of the catalyst design, in which several zeolites (e.g., Y, USY, ZSM-5) with different Si/Al ratios and textural characteristics were used as carriers for niobium (0.05 at%) following an Nb post-synthesis methodology. With the same aim, Nb-based Silicalite-1 was also prepared via a direct synthesis procedure. Data obtained from different characterization techniques allowed us to correlate the catalytic efficiency and catalytic properties and, based on this, to establish the main catalytic features of the developed materials able to lead to an optimum catalytic system for the oxidation of glucose and HMF toward SA or HMF toward FDCA. 

## 2. Results and Discussion

### 2.1. Catalysts’ Characterization 

The major difference between BEA and ZSM-5 on one side and H-USY on another side relates to the presence of microporosity in the formers and mesoporosity in the later. The two zeolites from the CBV series (i.e., H-USY CBV-600 and H-USY CBV-720) were prepared from H-USY through a steam and leaching treatment with a mineral acid. Thus, CBV-600 (Si/Al ratio of 5.2) was obtained by the steam treatment at 600 °C and CBV-720 (Si/Al ratio of 30.0) via a second steam treatment at 600 °C and leaching with mineral acids. The combination of steam treatment and acid leaching resulted in materials with disrupted crystal morphologies, larger mesoporosity (cracks and voids), and a progressively higher degree of dealumination [43,44]. 

Besides the textural changes, the presence of niobium species in the final catalysts led to changes in the density of Brønsted and Lewis acid sites. However, in the previously reported Nb-BEA zeolites, the N_2_ adsorption–desorption isotherms showed no effect on mesopores caused by the addition of niobium [45]. Accordingly, the decrease of the surface areas was not accompanied by changes in the pore size (estimated via BJH (Barret–Joyner–Halenda) method). A similar trend was also observed for Nb-Y5, Nb-Y30, and Nb-ZSM25 catalysts, in the actual study (Table 1).

**Figure 1:**
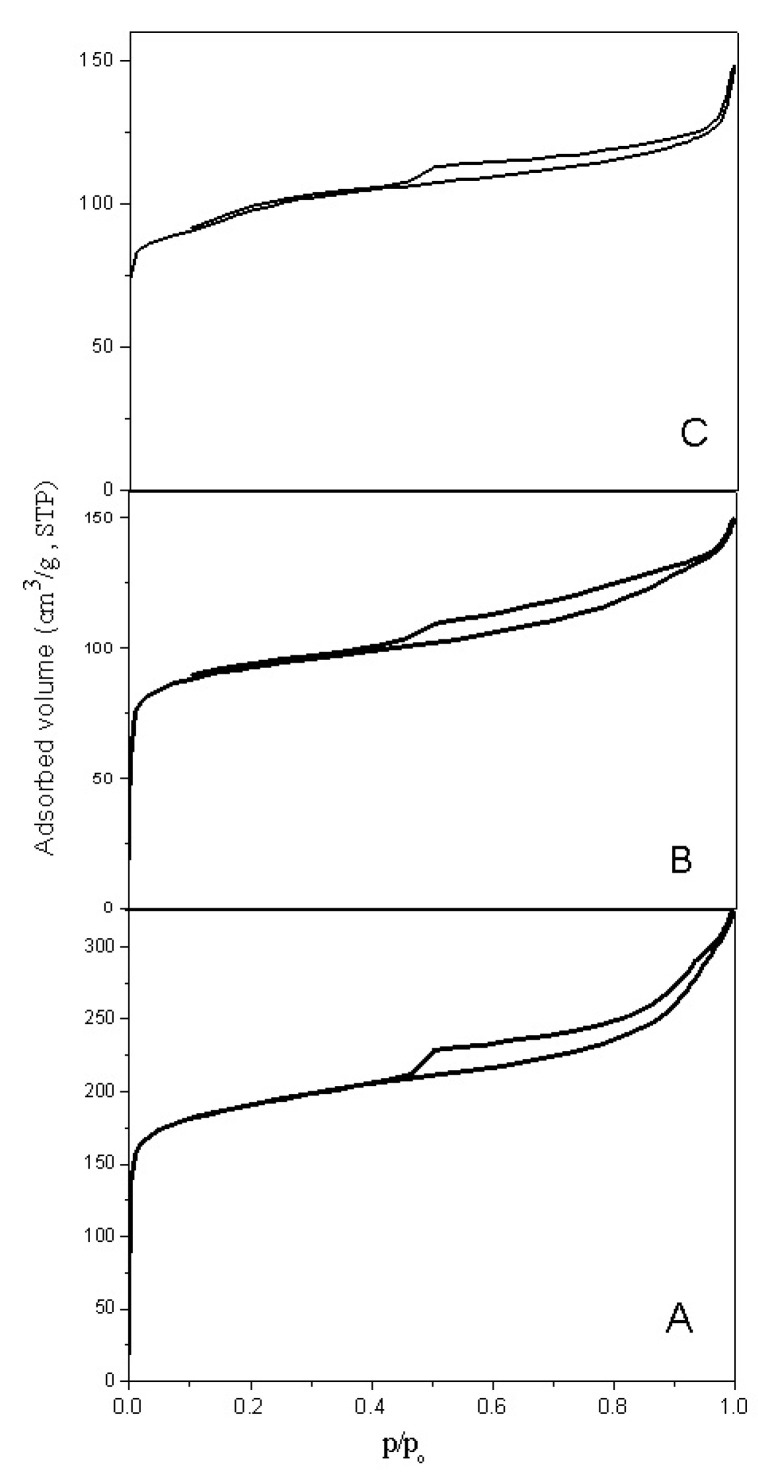
Nitrogen adsorption–desorption isotherms at 77 K for Nb-based zeolites: (**A**)—Nb-Y5, (**B**)—Nb-Y30, (**C**)—Nb-ZSM25.

The N_2_ adsorption–desorption isotherms (Figure 1) of Nb-based zeolites present a combination of Type I and Type IV isotherms, following the IUPAC classification [46], with the appearance of the micropore filling at low pressures (p/p_0_ < 0.1) and the hysteresis loops at higher pressures (p/p_0_ of 0.45–0.99), indicating a hierarchical porous system combining micro- and mesoporosity. Moreover, the isotherms also show a sharp rise in adsorbed amount near saturation (p/p_0_ of 1.0), associated with condensation in inter-particle voids (macropores). The macropores may be due to the dissolution of major amounts of aluminum during the first step of catalyst preparation. The pore size in the samples was estimated via the BJH (Barret–Joyner–Halenda) method (Table 1). 

**Figure 2:**
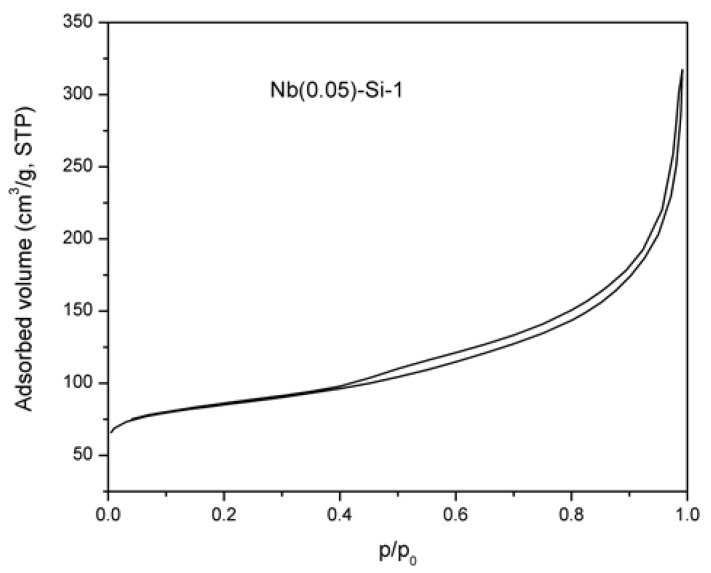
Nitrogen adsorption–desorption isotherms at 77 K for Nb-Si-1.

**Figure 3:**
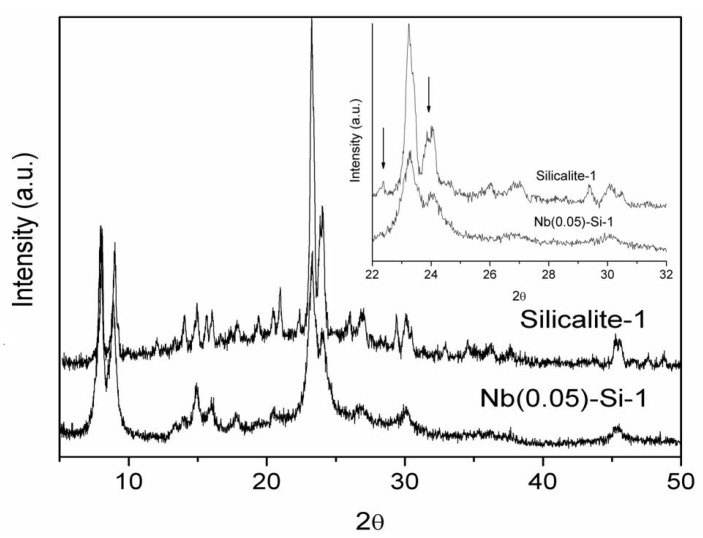
X-ray powder diffraction patterns of Silicalite-1 and Nb(0.05)-Si-1.

The N_2_ adsorption–desorption isotherm of Nb-Si-1 sample is given in Figure 2. Although the Nb-Si-1 sample showed a Type I isotherm, an hysteresis loop is observed, which is typically observed for the presence of mezopores. The sample displays a bimodal micro-mesoporosity (i.e., D_p_ = 1.9 and 3.5 nm) with a BET surface area of 290 m^2^ g^−1^ (Table 1, Figure 2). The BET surface area of the Nb-Si-1 is only slightly smaller than that of the pristine Silicalite-1 (415 m^2^/g). Most probably, this difference correlates to a decrease of the crystallinity of Nb-Si-1. Indeed, the XRD patterns (Figure 3) of Nb-Si-1 and pristine Silicalite-1 confirmed such a decrease of the crystallinity.

According to Prakash and Kevan [47], the very low intensity of the diffraction line at 22.3° (characteristic for Silicalite-1 structure) for the Nb-Si-1 sample and the existence of a single line at 24.1° instead of a doublet (observed for Silicalite-1) indicate the incorporation of Nb into the Silicalite-1 framework. No additional diffraction lines due an eventual presence of a crystalline Nb_2_O_5_ phase were observed, which is also in concordance with previous reports indicating that silica stabilizes amorphous niobia [48,49]. These confirm that Nb-silica catalysts prepared through sol-gel methods, using niobium ethoxide as a precursor, after calcination at 500 °C, contain Nb-O-Si linkages and superficial niobia in strong interaction with the support. Further calcination at temperatures higher than 900 °C induces diffraction lines characteristic to a TT-niobia phase [49,50].

**Figure 4:**
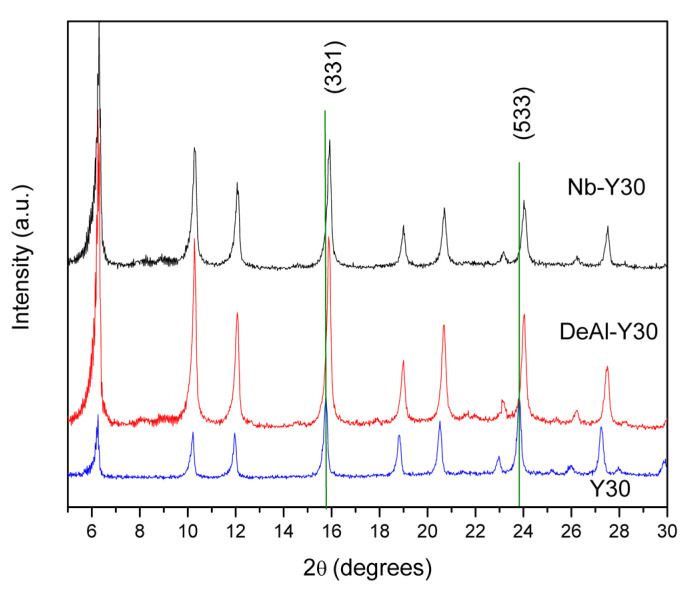
X-ray powder diffraction patterns of pristine H-USY CBV-600 (Y30), dealuminated H-USY CBV-600 (DeAl-Y30) and inserted Nb in dealuminated H-USY CBV-600 (Nb-Y30) samples (green lines help for the visualization of the shift of reflections (331) and (533)).

XRD patterns of Y30, DeAl-Y30 and Nb-Y30 samples are illustrated in Figure 4. They depict very intense X-ray lines in the large angle region of 5–50°, confirming the Y zeolite topologies (standard data of JCPDS No: 39–1380).

The zeolites crystallinity is well preserved after dealumination and Nb insertion. However, although the crystallinity is not affected, the shift of the reflections towards higher angle values indicates a decrease of the unit cell parameter a_0_ for both DeAl-Y30 and Nb-Y30 samples (Figure 4). No X-ray lines characteristic to niobium species were evidenced and no increase of the unit cell parameter was observed after the Nb insertion step confirming its high dispersion on the zeolite surface. Most probably, this Nb remains incapsulated into the framework pores as Nb(V) nano-oxides.

Indeed, the removal of Al atoms through dealumination with nitric acid results in the shrinking of the total zeolite framework because of the different –Al–O– and –Si–O– bond lengths of 1.91 Å and 1.69 Å [51], respectively. However, the size of the Nb ions (Nb^5+^ 0.591 Å, Nb^4+^ 0.768 Å and Nb^3+^ 0.795 Å) and the length of the Nb-O bond (1.89 Å for tetracoordinated Nb(V) species in zeolite) do not fit with the size of Al^3+^ (0.239 Å) and the length of Si-O bond (typically 1.60–1.69 Å in zeolites) [52]. 

**Figure 5:**
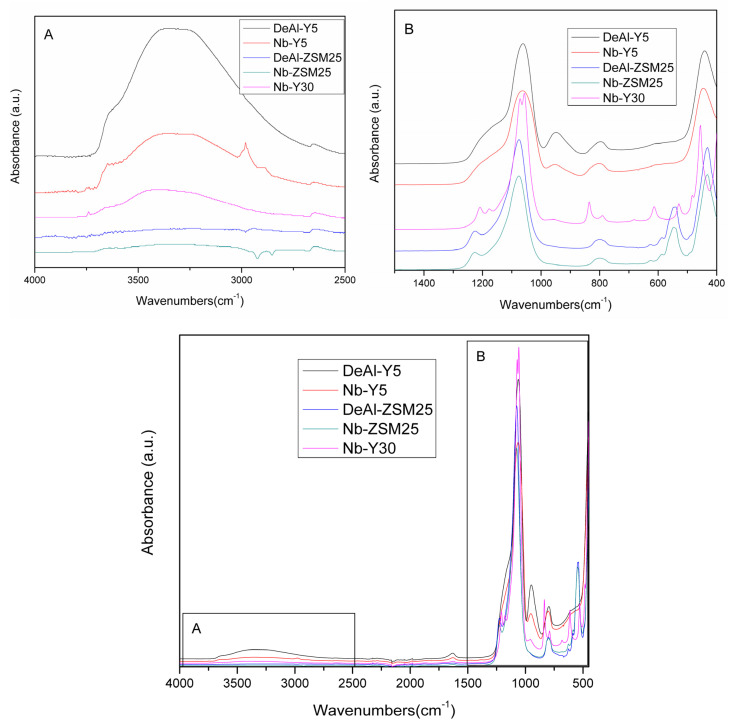
FTIR spectra of dealuminated Y5 (DeAl-Y5), Nb-Y30), ZSM-5 (DeAl-ZSM25) and Nb-ZSM25 samples with magnification on 4000–2500 (**A**) and 1500–400 cm^−1^ (**B**) regions.

These results are also confirmed by the FTIR spectroscopy results which suggest that the most probable state of niobium is that corresponding to Nb(V) in Nb(V)O-H species (Figure 5). The IR spectrum of dealuminated Y5 (DeAl-Y5) zeolite is characterized by the presence of a band at 3644 cm^−1^, assigned either to the Si-O(H)-Al [21] or to OH groups formed at the places left by the more easily removable framework aluminum atoms [53].

The second broad absorption band extending in a region between 3600–3200 cm^−1^ is due to Si-OH groups interacting with each other through H-bonds (Figure 5A). This large absorption band strongly suggests that the dealuminated Y5 sample is characterized by a high surface defectives. H-bonded SiO-H groups are also present in Nb-Y5 and Nb-Y30 samples but in a lower density compared to DeAl-Y5. In addition, the IR spectrum of these samples presents absorption at 3740 cm^−1^, most probably related to the presence of Nb(V)O-H groups [21]. 

The FTIR spectrum of DeAl-ZSM25 showed a different pattern. It does not contain clearly detectable isolated silanols but only a broad and very weak band in the 3600–3200 cm^−1^ range attesting a small population of silanol groups mainly interacting through H-bonding. In addition, no absorption bands attesting the presence of NbO-H species were evidenced for Nb-ZSM25. The absence of such bands can be explained by assuming an attachment of Nb(V) in the zeolite network, generating an excessive positive charge in the lattice, able to balance the charge of hydroxyl groups [54].

FTIR spectra in the 1500–400 cm^−1^ range (Figure 5B) do not offer concluding differences allowing for evidencing the presence of Nb-species in the zeolites frameworks. However, the zeolites structure is retained to a high degree after dealumination and Nb insertion, thus supporting the XRD measurements. 

**Figure 6:**
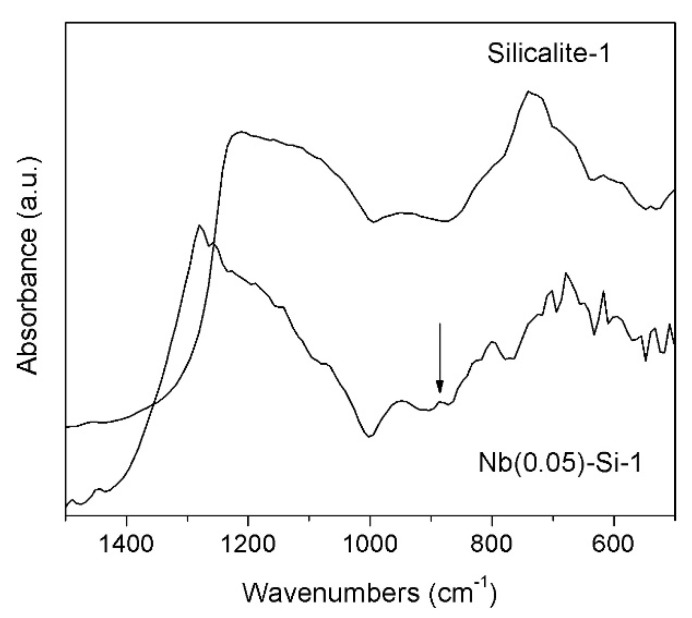
FTIR spectra of Silicalite-1 and Nb-Si-1 samples.

FTIR spectra of both Silicalite-1 and Nb-Si-1 samples are given in Figure 6. Except for a few characteristic bands, the spectra are similar. The FTIR spectrum of Nb-Si-1 showed no absorption bands at 3555–3602 cm^−1^ (not shown in Figure 6) confirming the absence of Nb (IV) in these materials, also in accordance with previous reports of Tielens et al. [55]. Similar to an Nb-Beta zeolite [21,45], the oxidation state of niobium is (V). However, contrary to the Nb-Beta zeolite [21,45], the band at around 884 cm^−1^ in the Nb-Si-1 sample indicates the presence of niobyl (–Nb = O) groups (by an arrow in Figure 6) [55], while the shift of the band at 1209 cm^−1^ (corresponding to the asymmetric stretching vibration of Si-O-Si) at 1280 cm^−1^ indicates the formation of new Si-O–Nb bonds. 

CO_2_- and NH_3_-TPD analyses revealed the acid–base properties of the Nb-Y5, Nb-Y30, Nb-ZSM25, and Nb-Si-1. The results compiled in Table 2 indicate that these catalysts contain a different population of acid and basic sites (mmol/g) depending on the zeolite nature, the dealumination degree, and the nature of the niobium species. The acidity of these catalysts also depends on the concentration of the vicinal aluminum T sites in the zeolites. While Brønsted acid sites associated with the vicinal aluminum T sites are lost, the residual Brønsted acidity is related to the concentration of the remaining isolated aluminum T sites and silanol groups [56]. The Lewis acid sites and cationic extra-framework aluminum species (AlO^+^) are formed during the dealumination step [56]. The residual extra-framework aluminum species also exhibit a strong acidity and prevent the solubilization of the zeolite framework in hot water. 

Zeolites with rich framework concentrations of aluminum were characterized by vicinal acid sites, where an AlO^+^/H^+^ exchange should be taken into consideration. In this case, the residual Brønsted acidity can be neutralized by the cationic extra-framework aluminum species, a feature already signaled by Lohse et al. [57]. 

As Table 2 shows, all samples display both acid and basic sites. If acidity can be generated by different species such as residual Al framework and extra-framework AlO_x_(OH) species, framework SiO-H groups, extra-framework isolated Nb(V)O-H, and Nb_2_O_5_ pore-encapsulated clusters, the only sites which may display a base character rather than an acidic one are the Nb-OH ones, as also suggested by several authors for niobia-silica mixtures [36,55]. The total basicity decreased in the following order: Nb-ZSM25 > Nb-Y5 > Nb-Y30 > Nb-Si-1, while the highest concentration of acid sites was measured for the Nb-Y5 sample. It is interesting to note that, for Nb-Si-1, the strength of basic sites is higher than in the others. The relative high desorption temperature of ammonia for the same catalyst can be correlated with the strength of niobium acid species (i.e., Nb = O or/and NbO-H). Finally, the base/acid sites ratio decreases in the order Nb-Si-1 > Nb-ZSM25 > Nb-Y30 > Nb-Y5. 

Overall, these characterization results are in line with those previously reported [21,45] showing that a post-synthetic insertion of Nb in the zeolite matrix led to Nb-zeolite catalysts comprised of extra-framework isolated Nb(V) sites (corresponding to Nb(V)O-H species) and Nb_2_O_5_ pore-encapsulated clusters. Also important, they were also detected residual framework Al-acid sites with a strong acidity, which prevents the solubilization of the zeolite framework in hot water [45]. As a function of the Si/Al ratio, the dealumination process takes place in different degrees, generating acid sites with different strength and structures with monomodal mesoporosity. The direct synthesis procedure applied for the preparation of the Nb-Sil-1 sample led to tetrahedral species presenting the –Nb=O group and bimodal micro-mesoporosity, in concordance with literature reports [55,58]. The presence of niobium species generates both extra acid and basic sites. 

### 2.2. Catalytic Tests

#### 2.2.1. Catalytic Wet Oxidation (CWO) in Aqueous Phase

The products distribution of the catalytic wet oxidation (CWO) highly depends on the experimental conditions, the catalytic active phase characteristics, and the support nature, as recently demonstrated for the glucose CWO by Nb-BEA [21], and in 5-hydroxymethylfurfural (HMF) CWO, by CoO_x_- and MnO_x_(10 wt.%)-Fe_3_O_4_@SiO_2_ catalysts [10]. This dependency is a consequence of the instability of the primary products, their evolution in time and the simultaneous concurrency of different reaction pathways. 

**Scheme 1:**
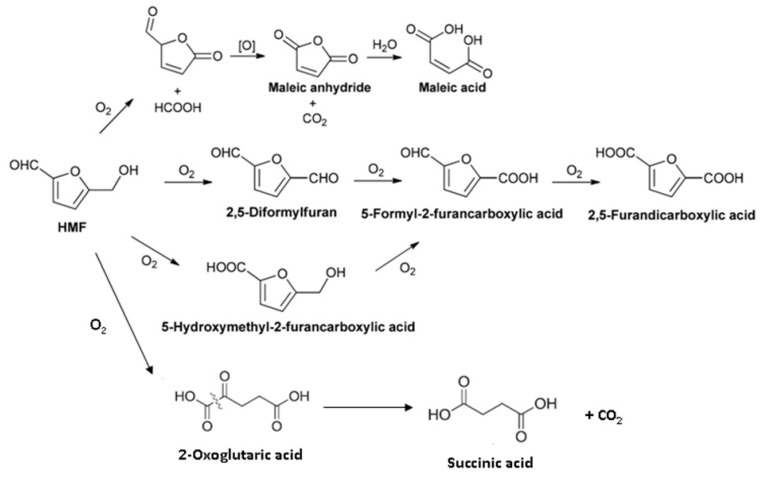
HMF oxidation MAc, FDCA, and SA [10].

The CWO of HMF may lead to different di-carboxylic acids such as maleic (MAc), furandicarboxylic (FDCA) and succinic (SA) acids (Scheme 1). While the formation of MAc takes place through the oxidative C–C bond cleavage and removal of two carbon atoms, SA can be formed via the 2-oxoglutaric acid intermediate (Scheme 1) [59] or through the oxidation of levulinic acid (LevA), possibly formed through the HMF hydration. Finally, the oxidation to FDCA involves the oxidation of both aldehyde and primary alcohol functions through intermediates as 5-hydroxymethylfuran-2-carboxylic acid (HMFCA), 2,5-diformylfuran (DFF), and 5-formyl-2-furancarboxilic acid (FFCA) [18]. 

A screening of the HMF wet oxidation reaction conditions in the presence of Nb-BEA18 zeolite catalyst showed that the reaction takes place preponderantly towards maleic (MAc) and/or succinic (SA) acids (Scheme 1 and Table 3) irrespective of the experimental conditions (i.e., reaction temperature, oxygen pressure, or reaction time). High amounts of levulinic acid (LevA) as the hydration product were also obtained while FDCA was not detected (Table 3). 

As Table 3 shows, at low reaction temperatures (80 °C) and high oxygen pressures (12–16 atm), the HMF conversions were smaller than 7% even after 12 h reaction time (entries 1–3). Almost no LevA product was formed, indicating a high stability of HMF to hydration at this temperature. Regarding the oxidation products, no SA but only low amounts of MAc (3–5%) and 5-HMFCA were detected. At higher temperatures (120 °C, entries 4–7) and lower oxygen pressures (4–10 atm), HMF starts to be transformed into LevA (15–37%) with a conversion of 15–52% along with MAc (31–43%). Except for the reaction at 10 atm (Table 3, entry 4) leading to a selectivity of 12.6% in SA, no other oxidation products were detected. As expected, the amount of MAc increased with the oxygen pressure, which is the reverse of LevA. At 140 °C and 10 atm molecular oxygen, HMF was totally converted after only 6 h resulting LevA (15.3%), SA (34.8%), and MAc (31.0%) as oxidation products (entry 9). Longer reaction times only have a small influence on the product distribution (12 h, entry 8). On the other hand, a further increase of the reaction temperature and the oxygen pressure produces higher amounts of SA, indicating a similar trend as observed previously for glucose CWO on Nb-BEA catalysts [21].

Reactions carried out at 140 °C and 10 atm of molecular oxygen, in the presence of Nb-Y5, Nb-Y30, and Nb-ZSM25, also led to three main reaction products: LevA obtained through the HMF hydration, oxidation of HMF/LevA to SA, and oxidation of HMF to MAc (through oxidative cleavage of C-C), irrespective of the catalyst or substrate (i.e., glucose or HMF) nature (Table 4). However, the distribution of these products is different as a function of the substrate nature and the catalyst characteristics.

**Figure 7:**
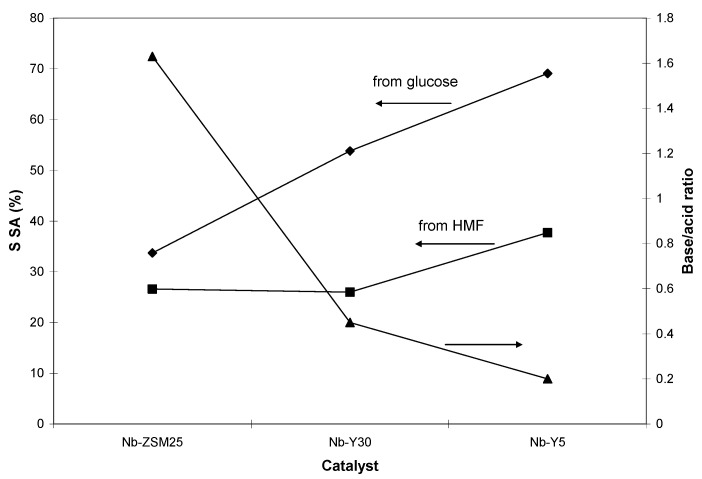
The variation of the SA selectivities as a function of the catalyst nature and the base/acid ratio.

Therefore, as Table 4 shows, the CWO of glucose leads to higher selectivities to SA than HMF, irrespective of the catalyst nature (entries 1, 3 and 5), but its magnitude is highly influenced by the base/acid site ratio in the catalyst (Figure 7). 

These results support the recently proposed mechanism for the production of SA through the CWO of glucose [21]. Accordingly, SA is produced through the synergetic participation of two kinds of active sites: (i) the extra-framework AlO_x_(OH) species which dehydrate the glucose to levulinic acid followed by (ii) its subsequent oxidation, onto the nano-oxide Nb_2_O_5_ particles, located in the zeolite channels (as extra framework species and/or partly framework penta-coordinated species). However, a higher amount of extra framework AlO_x_(OH) species is generated in the dealumination step of zeolites with low Si/Al. Therefore, the highest selectivity to SA in glucose CWO in the presence of the Nb-Y5 catalyst can be easily explained. Even more important, the involvement of Nb-OH species is clearly demonstrated in the oxidation step of glucose or in the directly oxidation of HMF. However, this mechanism seems to be an involved Nb-OH species with acid character since the lower the base/acid sites ratio, the higher the selectivity to SA is. In other words, a higher concentration of acid sites leads to a higher selectivity to SA. 

#### 2.2.2. Oxidation Reactions with Organic Peroxides

The presence of only very low amounts of the HMFCA intermediate and the lack of FDCA product suggest the Nb-based catalysts inability to oxidize the primary alcohol function of the HMF substrate (Scheme 1), at least in the CWO reaction conditions. The failure of the above attempts for the production of FDCA made us move from the WO conditions to a catalytic oxygen transfer methodology, which requires oxygen donors such as H_2_O_2_ or RO_2_H. Although more benign and, therefore, a preferable oxygen donor, H_2_O_2_, in combination with acetonitrile as a solvent, led to no oxidation products. A similar behavior has recently been reported by some of us in the HMF oxidation [60] and by Choudhary et al. [61] in the solvent-free oxidation of benzyl alcohol to benzaldehyde. Therefore, all reactions were made by using *t*-butyl hydroperoxyde (t-BOOH) as an oxygen transfer agent and the main obtained results are presented in Table 5. As Table 5 shows, under the used reaction conditions, Nb-zeolite based catalysts displayed from moderate (53–54%) to high (99.0%) HMF conversions.

After 12 h, the FDCA selectivity was less than 20%, irrespective of the catalyst nature. The best catalytic results were obtained after 24–48 h, in the presence of Nb-ZSM25 (61.3% FDCA selectivity for an HFM conversion of 96.7%) and Nb-Si-1 (63.8% FDCA selectivity for an HFM conversion of 99.0%) catalysts. Interestingly enough, the DFF intermediate was identified as only aleatory and in very low amounts. Accordingly, the reasonable reaction pathway for the HMF oxidation over these catalysts should follow the HMF → HMFCA → FFCA → FDCA sequence that is similar to that for supported Au and Pd catalysts [62] and for recently reported 10M@xNb@MNP (where M = Co, Mn and Fe) catalysts [60]. This route indicates a preferential adsorption of −CHO side chain of HMF, which requires the presence of basic sites and positive surface of the catalyst [63]. Differently to BEA and Y zeolites based catalysts, such conditions are properly supplied by Nb-ZSM25 and Nb-Si-1 which was characterized by the presence of both basic and acid sites at temperature the reaction is carried out (see the CO_2_- and NH_3_-TPD analysis results). Among the investigated catalysts, these displayed the highest base/acid sites ratio, with a domination of the basic sites (Table 3). These high basic OH^−^ ions catalyze the formation of geminal diols from aldehyde and water, while the Nb^+^ species catalyze the dehydrogenation steps, in accordance with a mechanism proposed by Donoeva et al. [63] for the HMF oxidation on gold nanoparticles supported on basic carbon materials and recently confirmed in our group for 10M@xNb@MNP (where M=Co, Mn, and Fe) catalysts [60]. 

It is also very important to note that these catalysts showed no significant loss in the catalytic activity during five recycling experiments, confirming their stability in the investigated reaction conditions. In addition, the ICP-OES analysis showed no metallic species in the reaction solution, also confirming the stability of the studied catalysts.

## 3. Materials and Methods 

### 3.1. Catalyst Preparation 

All the synthesis reagents were of analytic purity from Sigma-Aldrich and used as received. 

#### 3.1.1. Nb- Zeolite Catalysts Prepared through a Two-Step Post Synthesis Methodology

Since our previously work [21] indicated the BEA zeolite-based samples with 0.05 mol% Nb as the most efficient both for the glucose oxidation to succinic acid and for the direct conversion of glucose to HMF [45], in this work, we kept the same concentration for the catalysts synthesis. The used zeolites (purchased from ZEOLYST International Company) considered in this study had a Si/Al ratio of 30.0 (H-USY CBV-720), 12.0 (H-BEA 12), 18.0 (H-BEA-18), 25.0 (ZSM-5), 37.5 (H-BEA-37), and 5.2 (H-USY CBV-600), respectively. The Nb-BEA zeolite catalysts were synthesized as reported elsewhere [21] through a two-step post synthesis methodology, involving a dealumination of the zeolite with nitric acid followed by its impregnation with Nb ethoxide. A similar protocol was applied in this study and obtained catalysts were denoted as: Nb-ZSM25, Nb-Y5, Nb-Y30, Nb-Beta12, and Nb-Beta18, where the number represents the Si/Al ratio. 

#### 3.1.2. Nb-Silicalite Catalysts Preparation through the Sol-Gel Method 

The synthesis of NbSi-1 followed a reported procedure [46]. In agreement with this procedure, 10.8 mL of tetrabutylammonium hydroxide solution ((TBA)OH) was slowly added to 14.2 g of tetraethyl orthosilicate (TEOS) under stirring. In parallel, 1.7 mL of niobium ethoxide (corresponding to a final concentration of Nb of 0.05%mol) was slowly added to 10 mL of dry ethylic alcohol while stirring. The two solutions were then mixed by adding dropwise the niobium ethoxide solution to the TEOS-(TBA)OH solution, and the obtained mixture was stirred for 15 min. To this mixture, another 4.0 mL of aqueous (TBA)OH solution was slowly added. The final solution was heated to 70 °C under stirred, while water was added to compensate for the loss due to evaporation. The formed gel (60 mL) was then transferred into an autoclave and heated to 170 °C for 40 h without stirring. After crystallization, the product was separated from the mother liquor, washed with distillated water, and dried at 80 °C overnight. In the final step, the synthesized samples were calcined at 550 °C in air, for 20 h, to remove the organic template. 

Silicalite-1 sample was also prepared by applying a similar method as above except that the step for the addition of Nb was omitted. The obtained samples were designated as Si-1 and Nb-Si-1.

### 3.2. Catalyst Characterization

Adsorption–desorption isotherms of nitrogen at −196 °C, powder X-ray diffraction (XRD), Fourier transform infrared spectroscopy (FTIR), CO_2_- and NH_3_- temperature programmed desorption (NH_3_- and CO_2_-TPD), and inductively coupled plasma optical emission spectrometry (ICP-OES) have been previously applied to study the structure and composition of the bulk materials and were described elsewhere [21,45]. 

### 3.3. Catalytic Tests

#### 3.3.1. Catalytic Wet Oxidation (CWO) in Aqueous Phase

The activity tests in batch mode were carried out in a steel autoclave by adding 0.03 g of Nb- based zeolite catalyst to a solution of 0.5 mmoles of HMF in 10 mL of water. After closing, the reactor was pressured at 1–18 bars with molecular oxygen (purity ≥ 99.999% (*v/v*); Linde 5.0) and heated up to 80–180 °C, under stirring (1200 rpm), for 2–24 h. Parallel tests in the presence of glucose were also performed. After reaction, the oxygen was released, the catalyst was recovered by centrifugation, and the products separated by distillation under vacuum. 

#### 3.3.2. Oxidation Reactions with Organic Peroxides

Catalytic experiments were performed under vigorous stirring in a stainless steel autoclave (15 mL, HEL Instruments) under the following conditions: 50 mg HMF, 4.4 mL acetonitrile (ACN), 0.18 mL t-BuOOH (solution of 70%) and 25 mg catalyst were stirred at 120–140 °C, for 12–48 h. After reaction, the catalyst was recovered by centrifugation and the products separated by distillation under vacuum.

### 3.4. Products Analysis

The recovered products were silylated with 60 μL pyridine, 60 μL BSTFA (N,O-bis(trimethylsilyl) trifluoroacetamide), and TMCS (trimethylchlorosilane) silane agent, diluted with 1 mL of toluene and analyzed by GC-FID chromatography (GC-Shimadzu apparatus) equipped with a CPSIL8 column (0.53 mm × 5 μm × 25 m). All samples were analyzed in triplicates. The identification of the products was made by using a GC-MS Carlo Erba Instruments QMD 1000 equipped with a Factor Four VF-5HT column (0.32 mm × 0.1 μm × 15 m). 

The substrate conversion (C) and selectivities (S) to the reaction products were calculated from GC-FID chromatographic analysis by using the follow equations:

$$C\;=\frac{n_{i}\;-\;n{\;}_{t}}{n_{i}}\;\times \;100$$

where *n_i_*—initial moles of HMF used in reaction; *n_t_*—moles of untransformed HMF at time “*t*”, determined from GC analysis

$$S_{i}\;=\;\frac{Yield_{i}}{C}\;\times \;100$$

The recovered catalyst was dispersed in distilled water and centrifuged three times, and then dried at ambient temperature and used in a consecutive reaction of substrate.

## 4. Conclusions

In summary, Nb-zeolites based catalysts afford highly efficient catalytic systems for the oxidation of glucose and HMF to succinic acid or the oxidation of HMF to FDCA. The characterization results showed that, through the post-synthetic insertion of Nb in the zeolite matrix, the resulting Nb-zeolite catalysts possess a micro/mesoporous texture and comprise extra-framework isolated Nb(V) sites (corresponding to Nb(V)O-H species) and Nb_2_O_5_ pore-encapsulated clusters. Different amounts of extra framework AlO_x_(OH) species and residual framework Al-acid sites are also formed. The former species may stabilize the residual framework Al-acid sites preventing the solubilization of the zeolite framework in hot water. However, the geometry of Nb is clearly dependent on the synthesis method. Therefore, while dealumination followed by Nb insertion (i.e., Nb-Y5, Nb-ZSM25, Nb-Y30, Nb-BEA12 and Nb-BEA18) led to the above niobium species, the direct synthesis procedure (i.e., Nb-Sil-1) led to a bi-modal micro-mesoporous structure with tetrahedral niobium species presenting the –Nb=O group. 

CO_2_- and NH_3_-TPD measurements showed the presence of both acid and basic sites and the only sites which may display a base character rather than an acidic one are the Nb-OH sites. 

Catalytic experiments revealed the need for predominantly acidic sites for succinic acid synthesis under CWO reaction conditions, while the FDCA synthesis requires catalysts with predominantly basic sites in nature and organic peroxides as oxidation agents. In the same time, the presence of mesopores into the catalysts structure may be of help for the reactants and products diffusion, which may be the reason for the high catalytic activity.

## Figures and Tables

**Table 1:** Textural properties of the incorporated Nb in molecular sieves’ samples.

Catalyst	S_BET_, m^2^/g	V_p_, cm^3^/g	Pore Size, nm	Reference
BEA12	458	0.129	3.9 and 6.5	45
Nb-BEA12	391	0.104	3.9 and 6.4	45
BEA18	536	0.182	3.5	45
Nb-BEA18	438	0.114	3.5	45
Nb-Y30	624	0.297	3.9	This work
Y5	465	0.149	3.9 and 6.5	This work
Nb-Y5	303	0.127	4.0 and 6.5	This work
Nb-ZSM25	324	0.119	3.8	This work
Silicalite-1	415	0.150	-	This work
Nb-Si-1	290	0.077	1.9 and 3.5	This work

**Table 2:** Acid-base sites concentration determined through NH_3_- and CO_2_-TPD.

Sample	Acid Sites Concentration (mmols NH_3_/g Catalyst)	Base Sites Concentration (mmols CO_2_/g Catalyst)			Base/Acid Ratio
		100–170 °C	190–250 °C	Total	
Nb-Y5	0.235 (133 °C)	0.046	0.001	0.047	0.20
Nb-Y30	0.085 (99 °C)	0.035	0.003	0.038	0.45
Nb-ZSM25	0.043 (116 °C)	0.070	-	0.070	1.63
Nb-Si-1	0.016 (135 °C)	0.001	0.026	0.027	1.69

**Table 3:** The variation of the HMF conversion (C, %) and selectivites (S, %) to the main reaction products in the aqueous oxidation of HMF on the Nb-BEA18 zeolite catalyst.

Entry	Reaction Temperature, °C	P_O2_, atm	C, %	S, %			
				LevA	SA	MAc	HMFCA
1	80	16	6.9	0	0	4.6	2.8
2	80	14	4.0	0	0	2.8	3.5
3	80	12	3.8	1.7	0	2.8	4.6
4	120	10	52.1	15.1	12.6	43.5	0
5	120	8	42.9	24.7	0	42.9	0
6	120	6	44.7	29.0	0	37.4	0
7	120	4	15.0	37.0	0	31.7	0
8	140	10	100	26.7	31.9	21.4	0.6
9 ^a^	140	10	100	15.3	34.8	31.0	0.3

Reaction conditions: 0.063 g (0.5 mmol) HMF, 0.03 g Nb-BEA18 catalyst (0.015 mmol Nb), 10 mL water, 12 h, 1000 rpm. ^a^—6 h. Note: the difference in selectivity till 100 is given by lactic, glycolic, and water-soluble condensation products.

**Table 4:** The variation of the HMF conversion (C, %) and selectivites (S, %) to the main reaction products in the aqueous oxidation of HMF on Nb-based zeolite catalysts.

Entry	Catalyst	Substrate	C, %	S, %			
				LevA	SA	MAc	HMFCA
1	Nb-Y30	glucose	100	6.5	53.8	4.9	-
2	Nb-Y30	HMF	100	17.3	26.0	30.9	4.3
3	Nb-Y5	glucose	100	2.1	69.1	20.1	2.5
4	Nb-Y5	HMF	100	38.8	37.7	6.5	-
5	Nb-ZSM25	glucose	100	15.4	33.7	7.8	-
6	Nb-ZSM25	HMF	96.0	27.8	26.6	25.5	-

**Reaction conditions:** 0.5 mmol substrate (HMF or glucose), 0.03 g catalyst (0.015 mmol Nb), 10 atm O_2_, 140 °C, 10 mL water, 12 h, 1000 rpm. Note: the difference in selectivity till 100 is given by lactic, glycolic, and water-soluble condensation products.

**Table 5:** The variation of the HMF conversion (C, %) and selectivities (S, %) to the main reaction products in the oxidation of HMF with *t*-butyl hydroperoxyde (t-BOOH) and in the presence of Nb-based zeolites catalysts.

Entry	Catalyst	Reaction Time, h	C, %	S_HMFA_, %	S_DFF_, %	S_FDCA_, %
1	Nb-BEA18	24	53.9	73.8	0	5.6
2	Nb-Y30	12	85.8	22.3	0	8.1
3		24	99.1	41.7	0.6	22.5
4	Nb-Y5	12	80.5	52.1	0	21.4
5		24	71.0	53.1	0	23.1
6	Nb-ZSM25	12	53.3	73.2	1.2	12.6
7		24	96.7	15.0	0	61.3
8	Nb-Si-1	12	95.0	47.3	0.6	15.2
9		24	99.0	47.0	0	26.0
10		48	99.0	9.2	0	63.8

**Reaction conditions:** 0.050 g (0.4 mmol) HMF, 0.025 g catalyst (0.0125 mmol Nb), 4.4 mL acetonitrile, 0.18 mL 70% *t*-BuOOH, 140 °C, 1000 rpm. Note: the difference in selectivity till 100 is given by lactic, glycolic, and succinic acid.
